# Hydrocephalus in pediatric posterior fossa tumors: predictors and outcomes from a single center in Latin America

**DOI:** 10.1007/s00381-026-07179-y

**Published:** 2026-03-03

**Authors:** Cleiton Formentin, Leo Gordiano Matias, Lucas de Souza Rodrigues dos Santos, Carlos Eduardo Vasconcelos Miranda, Helder Tedeschi, Andrei Fernandes Joaquim, Enrico Ghizoni

**Affiliations:** 1 Department of Neurosurgery, Boldrini Children’s Hospital, Cidade Universitária, 1270 Dr. Gabriel Porto St., SP Campinas, 13083-210 Brazil; 2 Department of Neurology, University of Campinas, Campinas, Sao Paulo, Brazil

**Keywords:** Hydrocephalus, Infratentorial tumors, Posterior fossa tumors, Shunt insertion

## Abstract

**Background:**

The management of hydrocephalus in pediatric posterior fossa tumors remains highly variable. This study aims to analyze hydrodynamic factors and identify predictors of CSF diversion procedures in children undergoing posterior fossa tumor resection.

**Methods:**

A retrospective cohort study was conducted with pediatric patients who underwent posterior fossa tumor resections at a single center in Latin America. Poisson regression models were applied to analyze associations with ventriculomegaly, need for cerebrospinal fluid (CSF) diversion, postoperative hydrocephalus, and shunt dependence. Survival outcomes were assessed using Kaplan–Meier curve analysis.

**Results:**

The study analyzed 135 pediatric patients with posterior fossa tumors. Preoperatively, 71.85% had ventriculomegaly and 36.29% required CSF diversion. Postoperatively, 16.32% required new CSF diversion, and 25.93% remained shunt dependent. Multivariate analysis revealed that younger age, quadrigeminal cistern involvement, and metastasis were predictors of shunt requirement. Pilocytic astrocytomas were less prevalent among patients with hydrocephalus, while PNET/ATRT were more common. Postoperative hydrocephalus was linked to solid tumors, midline location, and metastasis. “Other embryonal tumors” had a 1.4 times higher likelihood of requiring a VPS postoperatively. The degree of tumor resection was not significantly associated with postoperative hydrocephalus. Considering shunt dependence, patients with ependymomas had a 1.56 times higher likelihood of persistent hydrocephalus. The 5-year OS rate was lower in patients with hydrocephalus (69.4% *vs* 90.7%).

**Conclusion:**

Tumor resection alone may not suffice to prevent hydrocephalus, particularly in younger children and those with ependymomas. High shunt dependency and related complications highlight the importance of early identification and careful patient selection for CSF diversion.

**Supplementary Information:**

The online version contains supplementary material available at 10.1007/s00381-026-07179-y.

## Introduction

Between 45 and 60% of pediatric tumors are located in the posterior fossa [1, 2]. Regardless of histological classification, these patients often present with symptoms of increased intracranial pressure (ICP) due to hydrocephalus, a common cause of clinical deterioration at the time of diagnosis, which can lead to high rates of morbidity and mortality [3, 4].

Preoperatively, 80 to 90% of children with posterior fossa tumors exhibit ventriculomegaly on initial imaging [5–7]. Meanwhile, postoperatively, between 18 and 40% of patients develop hydrocephalus, requiring subsequent surgical procedures for cerebrospinal fluid (CSF) diversion, exposing these individuals to a second source of morbidity [6, 8–11]. There is considerable variation in the reported incidence rates of hydrocephalus in the literature, suggesting that different management protocols may lead to varying rates of shunt insertion.

The primary controversy in hydrocephalus management concerns whether it should be treated prior to the resection of the primary tumor. Some authors advocate for performing a permanent CSF diversion before definitive tumor removal, most commonly a ventriculoperitoneal shunt (VPS) [12, 13]. Conversely, others propose a temporary diversion procedure, such as an external ventricular drain (EVD), combined with corticosteroid therapy to control symptomatic hydrocephalus, suggesting that these procedures would decrease the morbidity and mortality of tumor resection [14, 15]. To reduce permanent shunt use and EVD-related complications, some authors advocate routine preoperative endoscopic third ventriculostomy (ETV) [16]. In one study, prophylactic ETV reduced postoperative hydrocephalus from 26.8 to 6% [16] However, this approach may expose about 70% of children to an unnecessary procedure with inherent risk [17].

Advances in surgical techniques and neurosurgical expertise have significantly reduced complications in posterior fossa tumor surgeries, but this has not been accompanied by a corresponding decrease in postoperative hydrocephalus rates [18]. This study aims to analyze the outcomes of hydrocephalus treatment and identify predictors of the need for CSF diversion procedures in pediatric patients undergoing posterior fossa tumor resection at a pediatric oncology referral center in Latin America, while also describing the lessons learned from our management approaches.

## Methods

The present study is a retrospective cohort analysis based on medical records of pediatric patients who underwent surgical resection of posterior fossa tumors at Boldrini Children’s Hospital (Campinas, São Paulo, Brazil).

### Patient selection

The study enrolled patients under the age of 19 who underwent their first resection of a posterior fossa tumor at a single institution from January 2011 to January 2021. The start and end dates align with the period of care provided by the uniform neurosurgery team, ensuring a consistent and standardized approach to hydrocephalus management. Exclusion criteria comprised patients with insufficient medical records, a follow-up period shorter than 6 months, those previously operated on by a different neurosurgical team, and patients diagnosed with diffuse midline gliomas.

Three researchers, previously trained in its application, collected the data using a semi-structured form, which was subsequently double-checked. The research project was approved by the Ethics Committee of the Boldrini Children’s Hospital (CAAE: 71579317.7.0000.537).

### Data analysis

Demographic, clinical, and complementary examination data were collected from medical records at admission and throughout the follow-up period. MRI scans of the brain and neuroaxis were evaluated by the study’s researchers and neuroradiologists from the department. Evaluation included preoperative, immediate postoperative (within 72 h), and late control neuroimaging. Tumor volume was estimated using a modified ellipsoid formula ([*A* × *B *× *C*]/2), where *A*, *B*, and *C* represent the maximum diameter in centimeters of the tumor in each of the three dimensions. The lesion’s location was determined based on the primary anatomical structure involved. Lesions classified as midline primarily affected the fourth ventricle, cerebellar vermis, quadrigeminal cistern and/or brainstem. Histological data were obtained from reports issued by a single neuropathologist based on the WHO classifications of 2007 and 2016 [19, 20]. Metastasis included both diffuse leptomeningeal spread and secondary solid disease and was determined through CSF analysis (oncotic cytology) and neuroaxis MRI.

The degree of tumor resection and signs of tumor recurrence were assessed using immediate postoperative and late follow-up images, respectively. Surgical resection was categorized into three groups: total resection, near-total resection (≤ 1.5 cm^2^ of residual tumor), and subtotal resection (> 1.5 cm^2^) [21].

In this study, ventriculomegaly and hydrocephalus were treated as related but conceptually distinct entities. Ventriculomegaly was defined purely as a radiological finding, operationalized by an Evan’s Index greater than 0.30 on neuroimaging, irrespective of clinical status. This definition was used to describe ventricular enlargement at presentation and to explore its association with tumor-related anatomical factors. Hydrocephalus, in contrast, was defined as a clinical–physiological condition, reflecting disturbed CSF dynamics leading to symptomatic intracranial hypertension and requiring therapeutic intervention. Accordingly, hydrocephalus was considered present only when ventricular dilation was associated with clinical deterioration and resulted in a decision to perform CSF diversion (temporary or permanent).

This distinction was intentionally maintained throughout the analyses. Ventriculomegaly was analyzed as an imaging marker of ventricular enlargement, whereas hydrocephalus represented a clinically meaningful endpoint directly linked to treatment decisions. Shunt dependence was defined as persistent hydrocephalus requiring a permanent shunting device. New postoperative hydrocephalus was classified as early (within 30 days following surgical resection) or late (after 30 days).

**Fig. 1:**
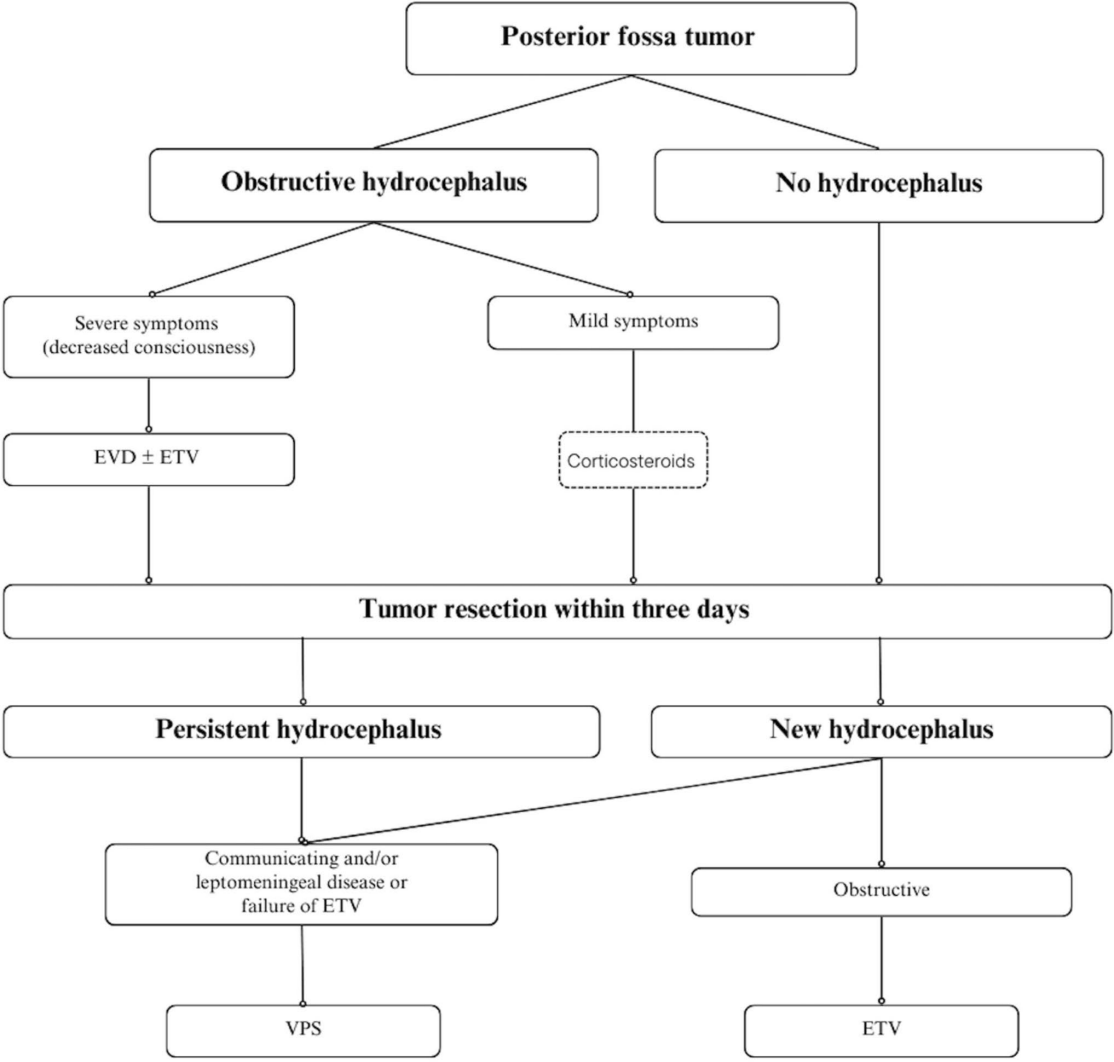
Flowchart depicting the management approach for pediatric patients with posterior fossa tumors and associated hydrocephalus in our center

The institutional strategy for managing hydrocephalus in children with posterior fossa tumors who presented primarily to our institution without prior CSF diversion is summarized in Fig. 1 and is described here to clarify clinical decision-making. All patients were initially stratified according to the presence of hydrocephalus and the severity of clinical symptoms. Patients presenting without hydrocephalus underwent early tumor resection without prophylactic CSF diversion. In patients with obstructive hydrocephalus, management was guided by neurological status. Those with severe symptoms or impaired consciousness underwent immediate temporary CSF diversion, most commonly EVD, with or without ETV, followed by tumor resection as soon as clinically feasible, typically within three days.

Clinically stable patients with obstructive hydrocephalus and mild symptoms were initially managed with corticosteroids and close monitoring, proceeding directly to early tumor resection without routine preoperative diversion. Patients referred from other centers after prior CSF diversion were managed on an individual basis according to their clinical status and prior interventions, recognizing the heterogeneity of pre-referral management.

### Statistical analysis

Statistical analysis was conducted using the Statistical Package for Social Sciences program (IBM SPSS Statistics, v20.0; IBM Corp). A comparative analysis between groups was performed using the Chi-square test (categorical variables) and the t-test (continuous variables).

Poisson regressions were applied according to the dependent variables under study, namely (a) ventriculomegaly, (b) hydrocephalus requiring any diversion procedure, (c) new postoperative hydrocephalus, and (d) shunt dependence. Association measures were verified for each independent variable in the model. Regression model adjustments were made according to the Backward-Wald procedure. Only variables that reached a *p* value < 0.05 were considered statistically significant. The association measure of variables was verified through Prevalence Ratio (PR) values. Additionally, an alpha value of 0.05 and a 95% confidence interval (CI) were applied.

Overall survival (OS) and progression-free survival (PFS) analyses were conducted through Kaplan–Meier curve construction, with curve comparison performed by the log-rank test (Mantel–Cox). 

## Results

### Patient and tumor characteristics

The study included 135 patients with posterior fossa tumors, comprising 76 (56.30%) males. The median age of patients at diagnosis was 7.52 years (± 5.03), ranging from 0.18 to 18.83. The most frequently observed signs and symptoms in the cohort were headache (69.6%), nausea and/or vomiting (65.9%), gait disturbance (47.4%), and cranial nerve deficit (25.9%).

Topographically, most lesions affected the midline (70.37%), with an involvement of the fourth ventricle seen in 45 patients (33.33%). The main tumor volume was 35.21 cm^3^. Table 1 summarizes the baseline characteristics of the cohort.

**Table 1:** Baseline characteristics of the 135 patients in the cohort

Variable	*N*	%
Tumoral aspect		
* Solid-cystic*	67	49.62
* Solid*	59	43.70
* Cystic*	7	5.78
* Hemorrhagic*	2	1.48
Location		
* Fourth ventricle*	45	33.33
* Vermis*	35	25.92
* Cerebellar hemisphere*	30	22.23
* Brainstem*	26	19.25
* Quadrigeminal cistern*	11	8.15
* Cerebellopontine angle*	6	4.44
Tumor type		
* Pilocytic astrocytoma*	46	34.07
* Medulloblastoma*	37	27.40
* Ependymoma (WHO grade II)*	13	9.62
* Ganglioglioma (WHO grade I)*	6	4.44
* ATRT*	4	2.96
* Anaplastic ependymoma (WHO grade III)*	3	2.22
* Glioblastoma*	3	2.22
* Mature teratoma*	3	2.22
* Diffuse astrocytoma (WHO grade II)*	2	1.48
* Pinealoblastoma*	2	1.48
* Other PNETs*	2	1.48
* Dermoid/epidermoid cyst*	2	0.74
* Anaplastic ganglioglioma (WHO grade III)*	1	0.74
* Choroid plexus carcinoma*	1	0.74
* Subependymoma*	1	0.74
* Others*	5	3.70
Extent of resection		
* Total*	97	71.85
* Near-total*	11	8.14
* Subtotal*	24	17.77

### CSF dynamics

Ventriculomegaly was reported in 97 patients (71.85%), with 49 patients (36.29%) requiring a CSF diversion procedure. A total of 41 CSF diversion procedures (30.37%) were performed preoperatively, with 18 (13.33%) conducted prior to transfer to the referral hospital and the remainder performed upon arrival.

During hospitalization, the ventricular catheter was removed in nine patients (6.66%) who initially underwent a temporary EVD. Additionally, in one patient, due to a complication related to VPS, removal of the diversion system was possible without long-term neurological compromise. Furthermore, one patient experienced EVD failure, requiring a subsequent VPS. Finally, 35 patients (25.93%) remained shunt dependent.

**Fig. 2:**
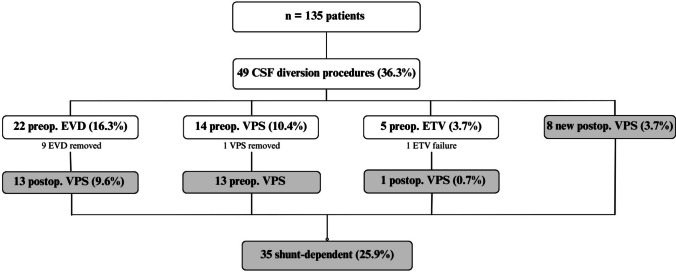
The flowchart illustrates the number of patients who underwent each CSF diversion procedure during specific periods

Eight patients (5.9%) developed new-onset postoperative hydrocephalus and required VPS. Of these, three procedures (2.22%) were performed in the early postoperative period: two due to residual lesions and one due to PICA ischemia causing fourth ventricle compression. Of the five patients (3.70%) who had VPS in the late postoperative period, hydrocephalus was associated with local recurrence in three cases and leptomeningeal disease in two cases. The flowchart in Fig. 2 summarizes the findings related to CSF diversion procedures.

Complications related to CSF diversion procedures occurred in 21 patients (42.86%), with infections being the most common, affecting 15 patients (30.61%). Among these, 10 underwent preoperative EVD (20.41%), 4 underwent direct VPS (8.16%), and 1 underwent ETV (2.04%)—the only complication associated with ETV in this cohort. Additionally, 7 of these patients (14.28%) had undergone external CSF diversion before being transferred to our institution. Other complications included one case of upward herniation (2.04%) and one case of peritoneal metastasis (2.04%) from an anaplastic ganglioglioma via VPS.

A stratified analysis comparing patients managed entirely at our institution during the preoperative period with those referred after prior CSF diversion revealed no statistically significant differences in demographic characteristics, tumor features, or shunt-related complications (Supplemental Table 1). Although CSF infection occurred more frequently among patients who underwent preoperative EVD placement at outside institutions, this association did not reach statistical significance in univariate analysis.

### Ventriculomegaly

The mean age of patients with preoperative ventriculomegaly (*n* = 97) was 7.03 years, compared to 8.39 years in those without, with no statistically significant difference between the groups. Patients with preoperative ventriculomegaly had longer hospital (*p* = 0.002) and ICU (*p* = 0.01) stays. The mean tumor volume was higher among patients with ventriculomegaly (*p* = 0.001, 95% CI 1.07–1.28). Regarding lesion topography, in the multivariate analyses, only fourth ventricle (*p* = 0.001, 95% CI 1.15–1.66) and quadrigeminal cistern tumors (*p* = 0.0001, 95% CI 1.24–1.56) were statistically significant for ventriculomegaly. Patients with metastases had a 1.4 times higher chance of presenting with ventriculomegaly (*p* = 0.0001, 95% CI 1.21–1.65).

When analyzing the three most frequent histological types, ventriculomegaly was less prevalent among patients with pilocytic astrocytoma (*p* = 0.03, 95% CI 0.57–0.97) but more prevalent among those with medulloblastoma (*p* = 0.0001, 95% CI 1.16–1.67) and ependymoma (*p* = 0.038, 95% CI 1.14–1.62). Table 2 presents the findings associated with ventriculomegaly.

**Table 2:** Results of the univariate and multivariate logistic regression analyses for the cohort related to preoperative ventriculomegaly

Variables	No. of patients with ventriculomegaly (%) [Mean ± SD]	Univariate *p* value	PR (95% CI)	Multivariate *p* value
Age				
* 0.19–6.95* * 7.10–18.83*	48 (70.6%) 49 (73.1%)	0.74	0.96 (0.78–1.19) -	0.74 -
Symptoms onset to diagnosis				
* 0–57 days* > *58 days*	52 (75.4%) 45 (68.2%)	0.35	1.11 (0.89–1.37) Ref	0.36 -
Length of hospital stay (days)	[9.78 ± 16.20]	**0.03**	1.01 (1.00–1.01)	**0.002**
Length of stay in the ICU (days)	[4.63 ± 8.99]	**0.01**	1.01 (1.00–1.01)	**0.01**
Tumor volume	[3.75 ± 1.21]	**0.001**	1.17 (1.07–1.28)	**0.001**
Tumor aspect				
* Solid-cystic* * Solid* * Hemorrhagic* * Cystic*	51 (76.1%) 40 (67.8%) 1 (50%) 5 (71.4%)	0.67	1.06 (0.65–1.73) 0.95 (0.57–1.57) 0.70 (0.16–3.02) Ref	0.79 0.84 0.63 -
Tumor location				
* Midline* * Outside midline*	70 (73.7%) 27 (67.5%)	0.46 -	1.17 (0.90–1.52) Ref	0.21 -
* CPA*	2 (33.3%)	**0.03**	0.44 (0.14–1.35)	0.15
* Cerebellar hemisphere*	19 (63.3%)	0.24	0.86 (0.64–1.14)	0.30
* Cerebellar vermis*	27 (77.1%)	0.41	1.11 (0.89–1.37)	0.35
* Fourth ventricle*	40 (90.9%)	**0.001**	1.38 (1.15–1.66)	**0.001**
* Brainstem*	12 (46.2%)	**0.001**	0.85 (0.41–1.00)	0.05
* Quadrigeminal cistern*	11 (100%)	**0.03**	1.39 (1.24–1.56)	** < 0.001**
Metastasis	25 (92.6%)	**0.007**	1.41 (1.21–1.65)	** < 0.001**
Tumor pathology				
* Ependymoma* * Ganglioglioma* * Medulloblastoma* * Pilocytic astrocytoma* * PNET/ATRT*	15 (93.8%) 4 (57.1%) 35 (89.7%) 27 (58.7%) 6 (100%)	** < 0.001**	0.64 (0.43–0.95) 1.46 (0.96–2.20) 0.89 (0.42–1.88) 1.56 (1.05–2.30) 0.91 (1.58–1.45)	**0.02** 0.07 0.76 0.27 0.70
Pilocytic astrocytoma	27 (58.7%)	**0.01**	0.75 (0.57–0.97)	**0.03**
Medulloblastoma	35 (89.7%)	**0.003**	1.39 (1.16–1.67)	** < 0.001**
Ependymoma	15 (93.8%)	**0.03**	1.36 (1.14–1.62)	**0.001**

*CI*, confidence interval; *PR*, prevalence ratio; *Ref*, reference; *SD*, standard deviation

### Hydrocephalus requiring CSF diversion procedure

The mean age of patients with hydrocephalus was 6.06 years, compared to 8.33 years for patients without hydrocephalus. In the adjusted model, younger children had a higher likelihood of requiring a CSF diversion procedure (*p* = 0.021, 95% CI 1.021–1.29). The interval between symptom onset and diagnosis was shorter for patients with hydrocephalus (*p* = 0.006, 95% CI 1.06–1.41), indicating faster symptom progression. Hospital and ICU stays were also longer for these children. Regarding symptoms, only altered consciousness (*p* = 0.0001, 95% CI 1.16–1.54), cranial nerve deficits (*p* = 0.0001, 95% CI 2.35–3.83), and seizures (*p* = 0.002, 95% CI 1.30–3.34) were statistically significant predictors of the need for a CSF diversion procedure in the multivariate analyses.

Regarding tumor characteristics, no patient with a hemorrhagic tumor at admission required a CSF diversion procedure (*p* = 0.04). Univariate analysis showed that fourth ventricle (*p* = 0.02) and quadrigeminal cistern tumors (*p* = 0.009) were significantly associated with hydrocephalus, while they were less prevalent in patients with cerebellar hemispheric tumors (*p* = 0.003). In the multivariate analysis, only quadrigeminal cistern tumors maintained a significant association with hydrocephalus (*p* = 0.02, 95% CI 1.03–1.50).

Patients with metastases also had a higher chance of requiring a CSF diversion procedure (*p* = 0.01, 95% CI 1.14–2.73). Regarding tumor type, pilocytic astrocytomas were less prevalent among patients with hydrocephalus, while PNET/ATRT were more common. When analyzing the three most frequent histological types individually, only those with pilocytic astrocytoma showed a statistically significant association, with a lower chance of requiring a procedure (*p* = 0.01, 95% CI 0.66–0.95). Table 3 summarizes these findings.

**Table 3:** Results of the univariate and multivariate logistic regression analyses for the cohort related to hydrocephalus requiring a CSF diversion procedure

Variables	No. of patients with hydrocephalus (%) [Mean ± SD]	Univariate *p* value	PR (95% CI)	Multivariate *p* value
Age				
* 0.19–6.95* * 7.10–18.83*	29 (42.6%) 20 (29.9%)	**0.01**	1.15 (1.02–1.29) -	**0.02** -
Symptoms onset to diagnosis				
* 0–57 days* > *58 days*	31 (44.9%) 18 (27.3%)	**0.03**	1.22 (1.06–1.41) Ref	**0.006** -
Length of hospital stay (days)	[7.65 ± 12.16]	**0.02**	1.01 (1.00–1.01)	**0.01**
Length of stay in the ICU (days)	[3.51 ± 6.49]	**0.01**	1.01 (1.00–1.01)	**0.01**
Signs and symptoms				
* Nausea/vomiting*	38 (42.7%)	**0.03**	1.10 (0.93–1.31)	0.27
* Headache*	32 (34%)	**0.04**	0.87 (0.75–1.01)	0.08
* Consciousness alteration*	10 (66.7%)	**0.001**	1.33 (1.16–1.54)	** < 0.001**
* Increased head circumference*	4 (100%)	**0.007**	3.00 (2.35–3.83)	** < 0.001**
* Seizures*	7 (70%)	**0.02**	2.08 (1.30–3.34)	**0.002**
Tumor volume	[3.64 ± 1.29]	0.4	1.03 (0.98–1.08)	0.19
Tumor aspect				
* Solid-cystic* * Solid* * Hemorrhagic* * Cystic*	28 (41.8%) 19 (32.2%) 0 (0.0%) 2 (28.6%)	**0.04**	1.09 (0.83–1.43) 1.04 (0.79–1.37) 0.78 (0.60–1.01) Ref	0.53 0.77 **0.04** -
Tumor location				
* Midline* * Outside midline*	38 (40%) 11 (27.5%)	0.11 -	0.97 (0.81–1.15) Ref	0.71 -
* CPA*	9 (52.9%)	0.30	0.86 (0.61–1.22)	0.41
* Cerebellar hemisphere*	4 (13.3%)	**0.003**	0.89 (0.73–1.10)	0.30
* Cerebellar vermis*	15 (42.9%)	0.34	0.93 (0.79–1.09)	0.36
* Fourth ventricle*	22 (50%)	**0.02**	1.03 (0.89–1.19)	0.71
* Brainstem*	8 (30.3%)	0.51	1.12 (0.92–1.36)	0.25
* Quadrigeminal cistern*	8 (72.7%)	**0.009**	1.24 (1.03–1.50)	**0.02**
Metastasis	15 (55.6%)	**0.02**	1.77 (1.14–2.73)	**0.01**
Tumor pathology				
* Ependymoma* * Ganglioglioma* * Medulloblastoma* * Pilocytic astrocytoma* * PNET/ATRT*	10 (62.5%) 2 (28%) 13 (35.1%) 8 (17.4%) 6 (100%)	**0.01**	1.14 (0.90–1.45) 1.00 (0.73–1.37) 0.93 (0.75–1.15) 0.82 (0.67–1.01) 1.40 (1.17–1.68)	0.28 1.00 0.49 **0.04** ** < 0.001**
* Others*	6 (42.9%)	**-**	Ref	-
Pilocytic astrocytoma	8 (17.4%)	**0.006**	0.79 (0.66–0.95)	**0.01**
Medulloblastoma	13 (35.1%)	0.95	0.88 (0.76–1.01)	0.8
Ependymoma	10 (62.5%)	0.65	1.17 (0.99–1.38)	0.73

*CI*, confidence interval; *PR*, prevalence ratio; *Ref*, reference; *SD*, standard deviation

### New-onset postoperative hydrocephalus

Among the eight patients who developed new-onset postoperative hydrocephalus (5.9%), statistical associations should be interpreted with caution due to the low number of events. Although solid tumors, midline location, metastatic disease, and “other embryonal tumors” (including PNETs and ATRTs) showed associations with postoperative shunt requirement, these findings are exploratory in nature and reflect limited statistical power rather than definitive predictors. In fact, all PNETs and ATRTs in the cohort required shunting at some point. No association was found between the three main histological types and postoperative hydrocephalus.

To assess the impact of clinically relevant perioperative variables on postoperative shunt dependency, we analyzed the association between preoperative shunt-related complications, including CSF infection, and the subsequent need for permanent shunting. Although CSF infection occurred more frequently among patients who became shunt dependent, this association did not reach statistical significance in univariate analysis and was not retained in the multivariate model (*p* > 0.05).

Although residual lesions were observed in four of the eight patients who required shunting postoperatively, the degree of tumor resection was not significantly associated with new postoperative hydrocephalus. Table 4 presents an overview of the data related to postoperative hydrocephalus.

**Table 4:** Results of the univariate and multivariate logistic regression analyses for the cohort related to post-tumor resection hydrocephalus

Variables	No. of patients with postoperative hydrocephalus (%) [Mean ± SD]	Univariate *p* value	PR (95% CI)	Multivariate *p* value
Age				
* 0.19–6.95* * 7.10–18.83*	5 (11.4%) 3 (6.0%)	0.35	0.66 (0.80–1.32) -	0.36 -
Tumor volume	[3.69 ± 1.28]	0.2	0.10 (−0.01–0.02)	0.72
Tumor aspect				
* Solid-cystic* * Solid* * Hemorrhagic* * Cystic*	3 (7,1%) 5 (11,1%) 0 (0,0%) 0 (0,0%)	**0.01**	1.06 (−0.77–0.01) 1.58 (−0.10–0.01) 0.45 (−1.70–1.71) Ref	0.78 **0.02** 0.98 -
Tumor location				
* Midline* * Outside midline*	8 (12.3%) 0 (0.0%)	**0.05** -	1.64 (−0.16–0.02) Ref	**0.03** -
* CPA*	0 (0.0%)	0.48	0.05 (0.01–0.18)	0.06
* Cerebellar hemisphere*	1 (3.7%)	0.29	0.04 (−0.18–0.01)	0.19
* Cerebellar vermis*	4 (16.7%)	0.09	0.58 (−0.14–0.03)	0.18
* Fourth ventricle*	3 (12.0%)	0.51	0.21 (−0.10–0.05)	0.51
* Brainstem*	2 (10.0%)	0.79	0.13 (−0.09–0.07)	0.75
* Quadrigeminal cistern*	0 (0.0%)	**0.03**	0.94 (0.01–0.07)	**0.004**
Metastasis	5 (12.0%)	**0.001**	1.14 (−0.27 to −0.01)	**0.03**
Leptomeningeal disease	3 (6.3%)	0.40	0.10 (−0.30–0.10)	0.10
Preoperative CSF infection	1 (7.1%)	0.61	0.66 (0.39–1.12)	0.13
Tumor pathology				
* Ependymoma* * Ganglioglioma* * Medulloblastoma* * Pilocytic astrocytoma* * PNET/ATRT*	2 (25.0%) 0 (0.0%) 3 (11.1%) 1 (2.6%) 1 (100%)	**0.01**	0.13 (−0.30–0.80) 0.10 (0.60–0.62) 0.50 (−0.12–0.01) 0.81 (−0.04–0.12) 1.40 (−0.69–0.70)	0.13 **0.001** 0.07 0.31 **0.001**
* Others*	0 (0.0%)		Ref	-
Pilocytic astrocytoma	1 (2.6%)	0.80	0.05 (0.01–0.12)	0.60
Medulloblastoma	3 (11.1%)	0.62	0.12 (0.10–0.45)	0.64
Ependymoma	2 (25.0%)	0.08	0.90 (−0.27–0.70)	0.30
Extent of resection				
* Total*	4 (5.7%)		0.10 (−0.07–0.24)	0.26
* Subtotal*	2 (20.0%)	0.18	0.04 (−0.13–0.22)	0.64
* Near-total*	1 (20.0%)		Ref	-

*CI*, confidence interval; *PR*, prevalence ratio; *Ref*, reference; *SD*, standard deviation

### Shunt dependent hydrocephalus

Univariate analysis showed younger age was associated with persistent hydrocephalus in shunt-dependent patients (*n* = 35), but multivariate analysis found no significant link between age and shunt dependency (*p* = 0.06, 95% CI 0.98–2.31), though a trend was observed.

Among the radiological variables, only tumor characteristics significantly influenced CSF shunt dependency, with solid and solid-cystic lesions being less prevalent in this group. In the analysis of all histological types, only ependymoma demonstrated a significant association with shunt dependency in both analyses (*p* = 0.0001, 95% CI 1.23–1.97), indicating that these patients had a 1.56 times greater likelihood of persistent hydrocephalus. Conversely, the degree of resection did not correlate with shunt dependency in either analysis. Table 5 details shunt-dependent hydrocephalus.

**Table 5:** Results of the univariate and multivariate logistic regression analyses for the cohort related to shunt-dependence

Variables	No. of patients			
	**Shunt dependents (%) [Mean ± SD]**	**Univariate *p* value**	**PR (95% CI)**	**Multivariate *p* value**
Age				
* 0.19–6.95* * 7.10–18.83*	24 (82.8.%) 11 (55.0%)	**0.03**	1.5 (0.98–2.31) -	0.06 -
Tumor volume	[3.75 ± 1.12]	0.3	0.96 (0.81–1.14)	0.64
Tumor aspect				
* Solid-cystic* * Solid* * Hemorrhagic* * Cystic*	19 (67.9%) 14 (73.7%) 0 (0,0%) 2 (100,0%)	**0.006**	0.68 (0.52–0.88) 0.74 (0.56–0.94) 0.45 (−1.70–1.71) Ref	**0.003** **0.02** 0.98 -
Tumor location				
* Midline* * Outside midline*	27 (71.1%) 8 (72.7%)	0.91 -	0.98 (0.64–1.48) Ref	0.91 -
* CPA*	0 (0.0%)	0.11	0.94 (0.12–1.09)	0.98
* Cerebellar hemisphere*	3 (75.0%)	0.87	1.05 (0.58–1.91)	0.86
* Cerebellar vermis*	9 (60.0%)	0.24	0.78 (0.50–1.23)	0.29
* Fourth ventricle*	17 (77.3%)	0.41	1.16 (0.82–1.65)	0.41
* Brainstem*	6 (75.0%)	0.81	1.01 (0.68–1.66)	0.80
* Quadrigeminal cistern*	6 (75.0%)	0.81	1.01 (0.68–1.66)	0.80
Metastasis	13 (86.7%)	0.12	1.34 (0.97–1.84)	0.07
Tumor pathology				
* Ependymoma* * Ganglioglioma* * Medulloblastoma* * Pilocytic astrocytoma* * PNET/ATRT*	10 (100.0%) 2 (100.0%) 8 (61.5%) 4 (50.0%) 5 (83.3%)	0.08	1.20 (0.83–1.19) 1.20 (0.84–1.71) 0.74 (0.83–1.72) 0.60 (0.01–1.01) 1.00 (0.27–1.30)	0.32 0.30 0.30 0.29 0.19
* Others*	6 (83.3%)		Ref	-
Pilocytic astrocytoma	4 (50.0%)	0.14	0.66 (0.32–1.35)	0.26
Medulloblastoma	8 (61.5%)	0.16	0.74 (0.45–1.21)	0.23
Ependymoma	10 (100.0%)	**0.02**	1.56 (1.23–1.97)	** < 0.001**
Extent of resection				
* Total*	21 (67.7%)		1.02 (0.85–1.51)	0.75
* Subtotal*	10 (83.3%)	0.32	1.50 (0.71–1.72)	0.73
* Near-total*	3 (75.0%)		Ref	-

*CI*, confidence interval; *PR*, prevalence ratio; *Ref*, reference; *SD*, standard deviation

### Survival outcomes

**Fig. 3:**
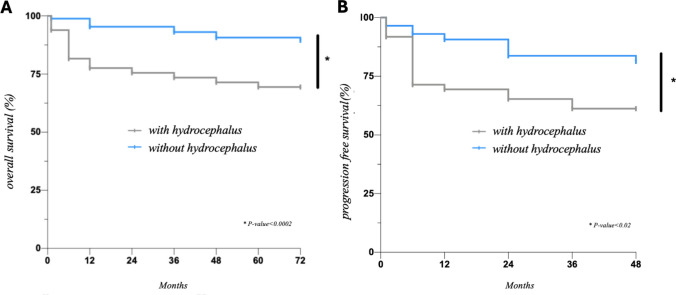
Kaplan Meier curves of overall survival (**A**) and progression free survival (**B**) among patients with and without hydrocephalus

Patients with hydrocephalus exhibited a 5-year OS rate of 69.4%, significantly lower than the 90.7% observed in patients without hydrocephalus (*p* < 0.0002). Additionally, these patients demonstrated reduced PFS compared to those without hydrocephalus (*p* < 0.02) (Fig. 3).

## Discussion

As survival rates improve among patients with posterior fossa tumors, hydrocephalus and treatment-related comorbidities have an increasingly significant impact on survivors’ quality of life. Despite numerous studies on the management of hydrocephalus in infratentorial tumors, no consensus has been established regarding the optimal approach [8, 9, 22, 23]. Although 71.9% of patients had ventriculomegaly, only one-third required CSF diversion. The hydrocephalus rate was 36.3%, with 25.9% shunt dependency; notably, 28.6% of shunted patients later became shunt independent. As many patients were referred from other centers, management protocols were not uniformly applied.

Although five patients underwent preoperative ETV in this cohort, the limited number of cases precludes any meaningful assessment of its effectiveness. In contrast, series from centers where ETV is routinely employed report variable but potentially favorable outcomes in selected patients with obstructive hydrocephalus. In a landmark study, Sainte-Rose et al. [16] reported a reduction in postoperative hydrocephalus requiring permanent shunting from approximately 25–30% to about 6% following preoperative ETV, suggesting a potential shunt-sparing effect in carefully selected cases. However, subsequent experience has shown that ETV outcomes are highly dependent on patient selection and institutional expertise. Our data therefore reflect a setting in which ETV was selectively used and should not be interpreted as an evaluation of its comparative efficacy.

Consistent with the Modified Canadian Preoperative Prediction Rule for Hydrocephalus (mCPPRH), our findings reinforce the importance of younger age, tumor type, and metastatic disease as relevant factors associated with the need for CSF diversion. However, unlike the mCPPRH—which was designed as a preoperative risk stratification tool—we did not attempt to construct, validate, or modify a predictive score. Instead, our analyses were exploratory and outcome-oriented, examining distinct endpoints related to CSF dynamics, including ventriculomegaly, hydrocephalus requiring CSF diversion, postoperative hydrocephalus, and shunt dependency, within a different clinical and healthcare context.

### Complications

Shunt placement carries the risk of complications, requiring lifelong monitoring. In our study, shunt-related complications occurred in 42.9% of cases, primarily due to infections (30.6%) in patients who underwent preoperative EVD at other hospitals prior to transfer. Vinchon and Dhellemmes [24] reported a lower infection rate of 13.6% in non-tumor hydrocephalus cases, suggesting that tumor-related factors may elevate risks. Children with infratentorial tumors often undergo multiple surgeries, intensive oncologic treatments, extended hospitalizations, and frequent clinic visits, which may contribute to higher infection rates. Patients with hydrocephalus had significantly lower survival rates, longer hospital and ICU stays, and poorer prognoses. However, this association may be influenced by a confounding bias, as more aggressive tumors tend to present with early hydrocephalus.

Shunt insertion in patients with posterior fossa tumors carries a risk of upward herniation, observed in one patient (2.04%) in our series, comparable to the 3% rate reported by Raimondi et al. [25] and lower than the 6% reported by Hoffman et al. [26]. Another concern is metastatic spread via shunt devices, with large autopsy series estimating an incidence below 0.5%, though its significance is debated [27, 28]. In our cohort, one patient (2.04%) developed peritoneal metastasis from an anaplastic ganglioglioma through a VPS, confirmed by pathology [29].

For this reason, we propose that patients presenting with posterior fossa tumors and hydrocephalus be promptly referred to specialized centers to optimize management and reduce infection-related complications.

### Demographic and clinical factors

Considering the high morbidity associated with shunt systems in general[30], it is deemed mandatory to select patients who will benefit the most from these procedures. Although preoperative ventriculomegaly was not significantly linked to age, younger children were more likely to require CSF diversion procedures and to remain shunt dependent. The average age of shunted patients (6.06 vs. 8.33 years) and the higher rate of shunt placement in those under 7 years old suggest that younger children are more susceptible to persistent hydrocephalus, consistent with findings from various case series [6, 8, 23, 31, 32]. Culley et al. [6] noted a more significant difference in shunt requirements for children under 3 years of age. However, when specifically evaluating patients who underwent CSF diversion procedures following tumor resection, as in the studies by Dias and Albright [9], there was no significant difference in age at diagnosis between those who required a shunt and those who did not.

Although rare in posterior fossa tumors, preoperative seizures were statistically significant for hydrocephalus, suggesting they may result from intracranial hypertension rather than local lesions or mass effect. Suri et al. [33] reviewed 511 patients with posterior fossa lesions, finding that 30 experienced seizures, although primarily in the postoperative period.

Considering this, younger age is consistently associated with shunt dependency across studies, a finding that was also confirmed in our cohort. Although rare, preoperative seizures should raise suspicion for intracranial hypertension [33].

### Tumor characteristics

While average tumor volume was associated with preoperative ventriculomegaly, it was not identified as a predictor for hydrocephalus or shunt dependency in this case series. In the cohort that underpinned the mCPPRH, Riva-Cambrin et al. [22] found no association between tumor size and the need for postoperative CSF diversion, even in univariate analysis. Consistent with this framework, tumor volume does not appear to be a determinant of persistent hydrocephalus.

Some studies have shown that tumors involving the midline are more likely to require CSF diversion procedures than tumors in the cerebellar hemispheres, likely due to their closer proximity to CSF circulation pathways [6, 23, 34]. However, other researchers have found no statistically significant difference related to tumor location, a result that is consistent with the findings of the present study [8, 9, 14]. This discrepancy may stem from differing criteria used to evaluate tumor location in imaging studies. When categorizing tumors as “midline” or “off-midline,” we found no significant associations with ventriculomegaly, hydrocephalus, or shunt dependency. However, in our analysis of new-onset postoperative hydrocephalus, a significant association with midline tumors (*p* = 0.03) was found, with a prevalence 1.64 times greater. Additionally, tumors in the quadrigeminal cistern were more likely to present with preoperative ventriculomegaly or require a diversion procedure, even in the postoperative period.

Metastases from posterior fossa tumors were significantly associated with ventriculomegaly (*p* = 0.0001) and hydrocephalus (*p* = 0.01), including in the postoperative period (*p* = 0.03). Lee et al. [35] identified Chang stages M3–M4 as independent predictors, while the Canadian cohort underlying the mCPPRH showed that metastases increased the risk of permanent CSF diversion by approximately 4.75 times [22]. 

In summary, our study demonstrated that tumor volume is not a reliable predictor of hydrocephalus or shunt dependency. Midline tumors—particularly those involving the quadrigeminal cistern—were associated with a higher risk of new postoperative hydrocephalus, while metastases remained strong independent predictors.

### Tumor type

The findings of this study are consistent with previous research on the prevalence of hydrocephalus in various tumor types. In particular, the multivariate analysis revealed that pilocytic astrocytomas are associated with a lower prevalence of hydrocephalus (*p* = 0.01, PR = 0.79), in agreement with the results of Bognár et al. [8], who reported the lowest need for CSF diversion among astrocytomas in posterior fossa tumors. Ependymoma was independently associated with shunt dependency among patients who initially underwent transient CSF diversion (*p* = 0.0001, PR = 1.56), a finding that is consistent with the Canadian cohort underlying the mCPPRH [22]. In that series, Riva-Cambrin et al. [22] identified ependymoma as a tumor type associated with a persistent risk of postoperative hydrocephalus requiring permanent CSF diversion.

Factors such as ventricular and cisternal involvement, combined with a higher prevalence in younger children, may contribute to the elevated rates of CSF diversion observed in ependymoma cases. Additionally, PNETs and ATRTs, categorized as “other embryonal tumors,” were significantly more likely to require diversion procedures (*p* = 0.0001, PR = 1.40), including in the postoperative period (*p* = 0.001, PR = 1.40), with all patients in this group ultimately requiring diversion.

The analysis of new-onset postoperative hydrocephalus must be interpreted within the context of a limited number of events. Only eight patients developed hydrocephalus after tumor resection, which restricts statistical power and increases the risk of type I error. Therefore, observed associations—particularly those related to tumor type and metastatic disease—should be viewed as exploratory and hypothesis-generating. While these signals are consistent with biologically plausible mechanisms and prior literature, they require validation in larger, preferably multicenter, cohorts before being used for clinical risk stratification.

Tumors in this cohort were classified according to WHO 2007/2016 criteria, reflecting the diagnostic standards and molecular data availability during the study period. The WHO 2021 classification introduces a molecularly driven framework that refines several entities represented in this series, with potential implications for subgroup interpretation. In particular, tumors previously grouped as “PNET” or broadly classified as embryonal tumors are now redefined into distinct molecular entities, such as ATRT and CNS embryonal tumor subtypes with specific genetic alterations. Similarly, posterior fossa ependymomas are now subdivided into molecular groups (e.g., PF-A and PF-B), which differ in age distribution, biological behavior, and prognosis. Adoption of WHO 2021 criteria could therefore further stratify the risk profiles observed in this study, especially within histological groups that demonstrated higher rates of hydrocephalus or shunt dependency.

In this context, patients with ependymoma—particularly those initially managed with temporary CSF diversion—have a high likelihood of requiring permanent shunting, which should be anticipated during treatment planning. Moreover, since all patients with “other embryonal tumors” required shunting, early identification and a proactive, aggressive approach to CSF diversion are essential.

### Extent of resection

We found no association between extent of resection and the need for postoperative CSF diversion, as restoring CSF flow does not always prevent the need for shunt placement [6–8, 23]. In the study by Culley et al.[6], 31% of patients with confirmed free CSF flow in the fourth ventricle following tumor removal developed postoperative hydrocephalus. The causes of persistent hydrocephalus after complete resection remain unclear, but it is thought that chronic blockage at the venous interface of the arachnoid granulations may hinder adequate CSF reabsorption [6]. In our series, three of the five patients who required shunting in the late postoperative period had hydrocephalus associated with local recurrence (*p* = 0.02), while two others were diagnosed with leptomeningeal dissemination, suggesting a link between postoperative hydrocephalus and metastases (*p* = 0.03). Thus, late hydrocephalus may arise not only in cases of local recurrence but also in distant metastatic contexts.

Furthermore, complete tumor resection often does not lead to an immediate decrease in ICP; instead, this typically occurs after a gradual “adaptation period” [36]. Bateman and Fiorentino [37] demonstrate that obstructive hydrocephalus in children is frequently associated with secondary venous compression, caused by direct tumor pressure on the venous sinuses—involving both intra- and extra-axial components. This process is thought to comprise the progressive reopening of subarachnoid spaces at the convexity, gradual restoration of Pacchionian granulation permeability, or redistribution of CSF into the spinal subdural space [37]. Younger children experience greater dural sinus compression than older children, which may explain higher rates of persistent hydrocephalus in this group.

In summary, postoperative hydrocephalus may occur despite restored CSF flow, with late-onset cases frequently associated with tumor recurrence or leptomeningeal dissemination.

### Limitations

This study has limitations. The relatively small sample size reduces statistical power and limits generalizability. As a retrospective review, it depends on historical records that may be incomplete or inconsistent, introducing potential bias. Limited molecular data prevented full WHO 2021 tumor classification, and the small number of ETV cases—due to delayed equipment availability—may have influenced the findings.

## Conclusion

Given the non-negligible rate of shunt-related complications observed in this cohort, particularly infections and long-term shunt dependency, careful selection of candidates for preoperative CSF diversion is essential. In this context, the prediction model proposed in this study is not intended to replace individualized clinical judgment or established decision-making processes. Rather, it serves as an adjunctive tool for structured risk stratification, systematizing routinely available clinical and imaging variables to identify patients more likely to benefit from early CSF diversion. Such an approach may be particularly valuable in borderline or equivocal cases and in referral centers managing heterogeneous populations, where management strategies vary widely. By supporting anticipatory planning and more consistent decision-making, risk-based stratification may help reduce potentially avoidable interventions and their associated morbidity while preserving the primacy of individualized clinical assessment.

Hydrocephalus in posterior fossa tumors is complex and warrants early referral to specialized centers. Younger age consistently predicts the need for CSF diversion, while tumor volume does not; risk is higher with midline lesions—especially quadrigeminal cistern—and with metastatic disease. Ependymoma often progresses to shunt dependence (notably after temporary diversion), and all “other embryonal tumors” in this series required shunting—supporting early identification and proactive CSF strategies. Postoperative hydrocephalus can arise despite restored CSF flow, frequently with recurrence or leptomeningeal spread; management should be individualized by tumor type, location, age, and disease course to minimize complications and preserve quality of life.

## Supplementary Information

Below is the link to the electronic supplementary material.

ESM1 SUPP. TABLE 1. Stratified analysis comparing patients managed entirely at the study center during the preoperative period with those referred after prior CSF diversion performed at outside institutions. (DOCX 16.4 KB)

## Data Availability

No datasets were generated or analysed during the current study.

## Abbreviations

ATRT: Atypical teratoid/rhabdoid tumor
CI: Confidence interval
CNS: Central nervous system
CPA: Cerebellopontine angle
CSF: Cerebrospinal fluid
ETV: Endoscopic third ventriculostomy
EVD: External ventricular drain
ICU: Intensive care unit
ICP: Intracranial pressure
MRI: Magnetic resonance imaging
OS: Overall survival
PFS: Progression-free survival
PICA: Posterior inferior cerebellar artery
PNET: Primitive neuroectodermal tumor
PR: Prevalence ratio
Ref.: Reference (used in tables)
SD: Standard deviation
VPS: Ventriculoperitoneal shunt
WHO: World Health Organization
