# Supplementary material for: Hydrocephalus in pediatric posterior fossa tumors: predictors and outcomes from a single center in Latin America
Source: Childs Nerv Syst. 2026 Mar 3;42(1):92. doi: 10.1007/s00381-026-07179-y (PMC12957642; doi:10.1007/s00381-026-07179-y)
Supplement: Supplementary file 1 — SUPP. TABLE 1. Stratified analysis comparing patients managed entirely at the study center during the preoperative period with those referred after prior CSF diversion performed at outside institutions. (DOCX 16.4 KB) [file 381_2026_7179_MOESM1_ESM.docx]

**SUPP. TABLE 1.** Stratified analysis comparing patients managed entirely at the study center during the preoperative period with those referred after prior cerebrospinal fluid (CSF) diversion performed at outside institutions.

|  | **No. of patients** |  |
| --- | --- | --- |
| **Variables** | **CSF diversion procedure at the study center (%)**  **[Mean ± SD]** | **Univariate p value** |
| Age |  |  |
| *0.19-6.95*  *7.10-18.83* | 13 (54.2%)  10 (58.8%) | 0.77 |
| Symptoms onset to diagnosis |  |  |
| *0-57 days*  *>58 days* | 14 (51.9%)  9 (64.3%) | 0.45 |
| Signs and Symptoms |  |  |
| *Nausea/vomiting* | 18 (58.1%) | 0.65 |
| *Headache* | 18 (62.1%) | 0.23 |
| *Consciousness alteration* | 5 (50%) | 0.65 |
| *Increased head circumference* | 2 (50%) | 0.8 |
| *Seizures* | 2 (28.6%) | 0.11 |
| Tumor volume | [3.75 ± 1.2] | 0.98 |
| Tumor location |  |  |
| *Cerebellar vermis* | 8 (72.7%) | 0.19 |
| *Fourth ventricle* | 11 (57.9%) | 0.83 |
| *Quadrigeminal cistern* | 3 (37.5%) | 0.24 |
| Shunt-related complications | 10 (50%) | 0.44 |
| CSF infection | 7 (50%) | 0.57 |

SD: standard deviation.
